# Long-term oncological results after transanal total mesorectal excision for rectal carcinoma

**DOI:** 10.1007/s10151-019-02094-8

**Published:** 2019-10-10

**Authors:** Jeroen C. Hol, Stefan E. van Oostendorp, Jurriaan B. Tuynman, Colin Sietses

**Affiliations:** 1 Department of Surgery, Gelderse Vallei Hospital, P.O. Box 9025, 6710 HN Ede, The Netherlands; 2 Department of Surgery, Amsterdam University Medical Center, Location VUmc, Cancer Center, Amsterdam, The Netherlands

**Keywords:** Colorectal cancer, Long-term results, Transanal TME

## Abstract

**Background:**

Transanal total mesorectal excision (TaTME) for mid and low rectal cancer has been shown to improve short-term outcomes, mostly due to lower conversion rates and with improved quality of the specimen. However, robust long-term oncological data supporting the encouraging clinical and pathological outcomes are lacking.

**Methods:**

All consecutive patients undergoing TaTME with curative intent for mid or low rectal cancer in two referral centers in The Netherlands between January 2012 and April 2016 with a complete and minimum follow-up of 36 months were included. The primary outcome was local recurrence rate. Secondary outcomes were disease-free survival, overall survival and development of metastasis.

**Results:**

There were 159 consecutive patients. Their mean age was 66.9 (10.2) years and 66.7% of all patients were men. Pathological analysis showed a complete mesorectum in 139 patients (87.4%), nearly complete in 16 (10.1%) and an incomplete mesorectum in 4 (2.5%). There was involvement of the CRM (< 1 mm) in one patient (0.6%) and no patients had involvement of the distal margin (< 5 mm). Final postoperative staging after neoadjuvant therapy was stage 0 in 11 patients (6.9%), stage I in 73 (45.9%), stage II in 31 (19.5%), stage III in 37 (23.3%) and stage IV in 7 (4.4%). The 3-year local recurrence rate was 2.0% and the 5-year local recurrence rate was 4.0%. Median time to local recurrence was 19.2 months. Distant metastases were found in 22 (13.8%) patients and were diagnosed after a median of 6.9 months (range 1.1–50.4) months. Disease-free survival was 92% at 3 years and 81% at 5 years. Overall survival was 83.6% at 3 years and 77.3% at 5 years.

**Conclusions:**

The long-term follow-up of the current cohort confirms the oncological safety and feasibility of TaTME in two high volume referral centers for rectal carcinoma. However, further robust and audited data must confirm current findings before widespread implementation of TaTME.

## Introduction

Transanal total mesorectal excision (TaTME) has the potential to lower the local recurrence rate after radical resection of mid and low rectal cancer. Currently available evidence shows an improvement in the quality of the surgical specimen and reduced number of R1 resections with longer distal margins in initial cohort studies [1–3]. Therefore, TaTME has the potential to lower the local recurrence rate after radical resection of mid and low rectal cancer. However, long-term data on local recurrence rates confirming encouraging pathological outcomes are lacking [4]. Over the past decades, adoption of total mesorectal excision (TME) as the surgical principle has reduced local recurrence rates and improved cancer-free survival rates [5]. Combined with neoadjuvant chemoradiation, the local recurrence rates have been reduced to 5% as demonstrated in a large randomized clinical trial [6].

Even though laparoscopic surgery has improved the short-term results of rectal cancer surgery, large randomized trials have shown that the oncological benefits are modest [6–8]. Laparoscopic TME is a difficult technique and this may negatively influence the results of surgery, especially as regards the lower part of the rectum In male patients with a small narrow pelvis, there is a limited space to mobilize the rectum with intact mesorectum.

In TaTME, the rectum is approached both from above and below, preferably at the same time [1]. Because the distal part of the rectum is approached from below, it is more accessible and the surgical planes are better visualized. The technique appears to be feasible and short-term outcomes seem promising in expert centers. However, the learning curve is steep, which might influence the results in low volume centers [3, 9]. Recently, the local recurrence rate after TaTME in Norway was reported to be 9.5% which led to a nationwide stop of TaTME and thorough investigation [10]. The results of the official investigations are eagerly awaited. Other single center series have reported local recurrence rates ranging from 2.3 to 5.7% with a median follow-up of 15–32 months [2, 11–15]. The aim of this study was to investigate the long-term oncological results after TaTME surgery in a large consecutive cohort from the two hospitals that started TaTME in The Netherlands with a minimum follow-up of 36 months.

## Materials and methods

### Patients

Between January 2012 and May 2016, all patients in the Gelderse Vallei Hospital, Ede, The Netherlands and Amsterdam UMC, location VUmc, Amsterdam, The Netherlands with histological proven distal or mid rectal carcinomas, who had elective TaTME, were included. Exclusion criteria were recurrent and/or locally advanced tumors with persistent threatened margins after neoadjuvant radiotherapy and palliative resections. Patients with curative resection of synchronous liver metastasis were included.

Preoperative assessment included magnetic resonance imaging (MRI) for local staging, computed tomography (CT) scan of the abdomen, CT scan or conventional X-ray of the chest to detect distant metastasis, and blood tests including serum carcinoembryonic antigen (CEA) levels. Each patient was discussed by a local multidisciplinary cancer board. Patients at medium risk, i.e., those with cT3b+ N0 or cT2–3 N1 tumors received preoperative radiotherapy with 5 Gy daily for five consecutive days. Patients with N2 disease or tumors with threatened margins (< 1.0 mm) to the mesorectal fascia were treated with chemoradiation therapy for 25 days with 2 Gy daily combined with administration of oral 5-fluorouracil.

The study was approved by the Ethics Committees of the participating centers. All procedures performed in studies involving human participants were in accordance with the ethical standards of the institutional and/or national research committee and with the 1964 Helsinki Declaration and its later amendments or comparable ethical standards.

### Surgical procedure

TaTME was performed as described previously [2]. The first patients were operated on by a single surgeon, performing both phases of the procedure sequentially. After the initial learning curve, the two team approach was introduced, with simultaneous abdominal and the transanal dissection. The splenic flexure was mobilized in the majority of the patients. Ligation of the inferior mesenteric vein was done near the pancreas.

The transanal phase consists of a thorough washout and the introduction of the anal platform; in the majority of the cases the GelPOINT Path Transanal Access Platform (Applied Medical, Rancho Santa Margarita, CA, USA) was used. In the first patients, a regular laparoscopic CO_2_ insufflator was used. In all other patients, the AirSeal insufflator (ConMed, Utica, NY, USA) was used. The purse-string location changed from the initial position directly behind the dentate line to a 3 cm higher position above the anorectal junction (if applicable for the location of the tumor, in proximal tumors it was placed below the tumor). Dissection was performed in a standardized fashion, starting the dissection dorsally and ventrally and thereafter dissecting the lateral plane. The abdominal and transanal team joined anteriorly. Specimen extraction was performed, after wound protection, through a Pfannenstiel incision. The anastomosis was preferably made side to end using a 31 EEA or 33 EEA hemorrhoid stapler (Medtronic, Dublin, Ireland).

### Data collection

Baseline data were collected regarding age, sex, American Society of Anesthesiologists (ASA) classification, body mass index (BMI), distance of the tumor from the anal verge, preoperative clinical staging and preoperative chemoradiation therapy. Pathological outcomes included pathological staging, macroscopic completeness of the resection, number of lymph nodes harvested and circumferential resection margin (CRM) involvement. All patients have had follow-up carried out according to the Dutch National Guidelines for Colorectal Cancer for a period of 5 years at the outpatient clinic. For this cohort, a full 36-month follow-up was available for all patients. Primary outcome was locoregional recurrence. Secondary outcomes included distant metastasis, disease-free and overall survival. Recurrent disease was defined as the presence of locoregional recurrence, distant metastases or death from rectal cancer.

### Statistical analysis

All data collection and statistical analysis were carried out using SPSS Statistics version 24 (IBM, Chicago, IL, USA). After analysis of numbers and percentages or median and range for each variable, a univariate binary regression analysis was performed for possible risk factors for local recurrence. Kaplan–Meier survival analysis was performed for local recurrence-free survival rates, disease-free survival rates and overall survival rates.

## Results

### Patient characteristics and clinical outcomes

From January 2012 to May 2016, a total of 159 consecutive patients underwent TaTME. 110 underwent surgery in Gelderse Vallei Hospital, Ede, The Netherlands, and 49 in Amsterdam UMC, location VUmc, Amsterdam, the Netherlands. Their mean age was 66.9 (10.2) years and 66.7% of all patients were men. The follow-up data for 36-month follow-up was complete for all patients. Neoadjuvant radiotherapy was administered in 112 patients (70.4%) and 117 received a primary anastomosis during surgery (73.6%). Thirty-nine patients (24.5%) encountered postoperative complications graded as Clavien–Dindo grade 3 or higher. Patient characteristics and short-term clinical outcomes are summarized in Table 1.

**Table 1:** Patient characteristics and clinical outcome

	*n* = 159
Sex	
Male	106 (66.7)
Female	53 (33.3)
BMI (mean) (± SD)	26.4 (4.3)
Age (years) (mean) (± SD)	66.9 (10.2)
ASA	
I	33 (20.8)
II	100 (62.9)
III	26 (16.4)
Height from AV (cm)	
Mean (± SD)	5.7 (3.5)
Median (range)	6 (0–15)
Height from AV < 4 cm	
Yes	47 (29.6)
Clinical tumor stage	
T1	2 (1.3)
T2	39 (24.5)
T3	103 (64.8)
T4	11 (6.9)
Tx	4 (2.5)
Clinical nodal stage	
N0	82 (51.6)
N1	47 (29.6)
N2	26 (16.4)
Nx	3 (1.9)
Synchronous metastasis	
M+	7 (4.4)
MRF threatened (before RT)	
No	125 (78.6)
Yes	34 (21.4)
Preoperative therapy	
RT	112 (70.4)
CRT	43 (27.0)
Anastomosis	
Primary anastomosis	117 (73.6)
End colostomy	42 (26.4)
Performed operation	
LAR TaTME	133 (83.6)
ISR/APE TaTME	26 (16.4)
Intraoperative complications	
Rectal perforation	2 (1.3)
Purse-string failure	1 (0.6)
Carbon dioxide (CO_2_) embolus	1 (0.6)
Postoperative morbidity	
No complications	46 (47.8)
Minor Clavien–Dindo 1–2	44 (27.7)
Severe Clavien–Dindo ≥ 3	39 (24.5)
Reoperation	36 (22.6)
Anastomotic leakage	10 (6.3)
Presacral abscess	14 (8.8)

Numbers in parentheses are percentages, unless mentioned otherwise

*BMI* body mass index (kg/m^2^), *SD* standard deviation, *ASA* American Society of Anesthesiologists, *cm* centimeters, *AV* anal verge, *MRF* mesorectal fascia, *RT* radiotherapy, *CRT* chemoradiotherapy, *LAR* low anterior resection, *ISR* intersphincteric resection, *APE* abdominoperineal excision, *Tx* or *Nx* means stage unknown based on preoperative MRI

### Oncologic outcomes

Pathological analysis showed a complete mesorectum in 139 patients (87.4%), nearly complete in 16 (10.1%) and an incomplete mesorectum in 4 (2.5%). There was involvement of the CRM (< 1 mm) in one patient (0.6%) and no patients had involvement of the distal margin (< 5 mm).

Pathological staging showed T0 in 13 patient (8.2%), T1 in 15 (9.4%), T2 in 74 (46.5%), T3 in 55 (34.6%) and T4 in 2 (1.3%). N stage was N0 in 118 patients (74.2%), N1 in 28 (17.6%) and N2 in 13 (8.2%). Final postoperative staging after neoadjuvant therapy according to the fifth AJCC classification was stage 0 in 11 patients (6.9%), stage I in 73 (45.9%), stage II in 31 (19.5%), stage III 37 (23.3%) and stage IV in 7 (4.4%).

**Fig. 1:**
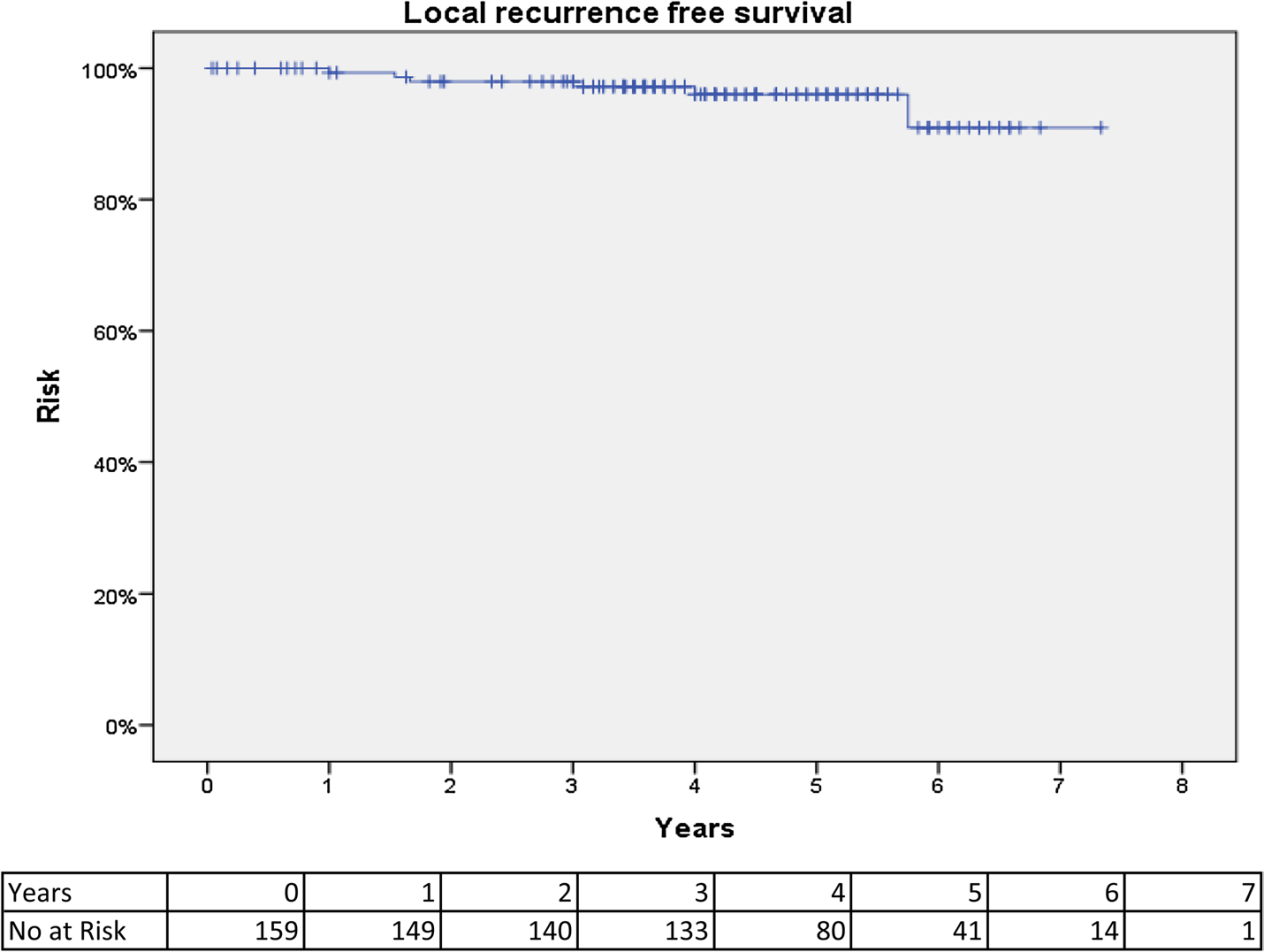
Kaplan–Meier curve of local recurrence-free survival after TaTME

The mean long-term follow-up was 54.8 months (range 36–88 months). The overall local recurrence rate was 3.8%, and median time to local recurrence was 19.2 months (range 5.9–30.0 months). Figure 1 shows a Kaplan–Meier (KM) curve of local recurrence. The local recurrence rate was 2.0% at 3 years and 4.0% at 5 years. An overview of all six cases of local recurrence and treatment can be seen in Table 2.

**Table 2:** Overview of cases with local recurrence

Surgery	p Stage	Complications	R	Neoadjuvant	Interval (months)	Location	Treatment	Survival
2012	T3N2	Presacral abscess	R0	Radiotherapy	18	Presacral	Palliative chemotherapy	57 months
2013	T2N1	None	R0	None	8	Presacral	Stoma and palliative chemotherapy	Alive, remission
2014	T3N0	Presacral abscess	R1	Chemoradiation	6	Presacral	Palliative treatment	12 months
2016	T3N0	Anastomotic leakage	R0	Chemoradiation	30	Presacral	APE	Alive
2014	ypT0N0	Presacral abscess	R0	Radiotherapy	19	Vesiculae	APE and debulking	Alive
2015	pT3N1	None	R0	None	27	Presacral	CRTX, exenteration	Alive

*APE* abdominoperineal excision, *CRTX* chemoradiation therapy

**Fig. 2:**
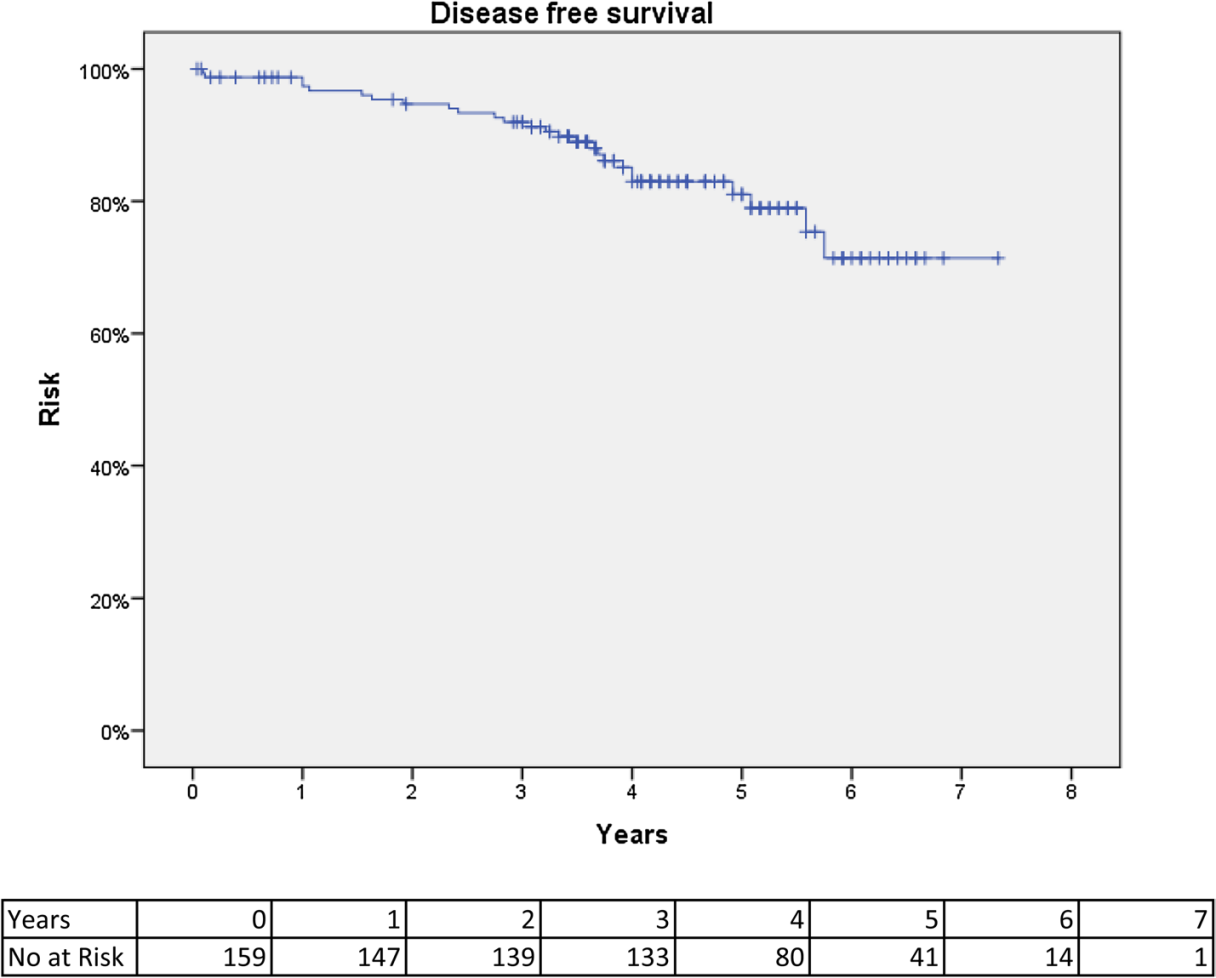
Kaplan–Meier curve of disease-free survival after TaTME

**Fig. 3:**
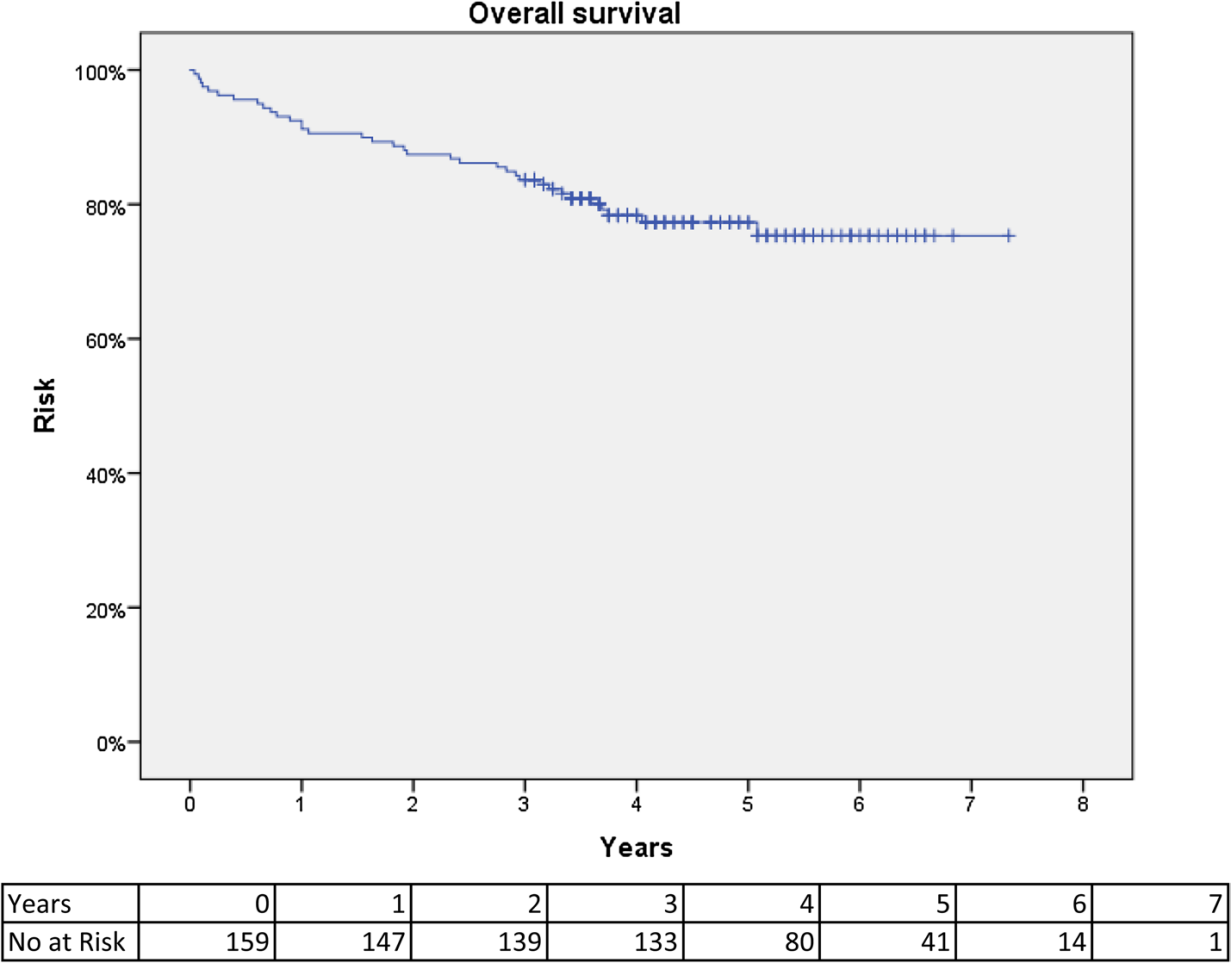
Kaplan–Meier curve of overall survival after TaTME

Disease-free survival was 92% at 3 years and 81% at 5 years. Figure 2 shows a Kaplan–Meier curve of disease-free survival. Distant metastases were found in 22 (13.8%) patients and were diagnosed after a median of 6.9 months (1.1–50.4). Two out of six patients with local recurrence had concomitant distant metastasis. Overall survival was 83.6% at 3 years and at 77.3% 5 years. Figure 3 shows a Kaplan–Meier curve of overall survival. The pathology results and long-term results are summarized in Table 3.

**Table 3:** Pathologic and long-term outcomes

	*n* = 159
Pathologic T-stage	
(y)pT0	13 (8.2)
(y)pT1	15 (9.4)
(y)pT2	74 (46.5)
(y)pT3	55 (34.6)
(y)pT4	2 (1.3)
Pathologic N stage	
N0	118 (74.2)
N1	28 (17.6)
N2	13 (8.2)
Quality of specimen (Quirke)	
Incomplete	4 (2.5)
Nearly complete	16 (10.1)
Complete	139 (87.4)
CRM+	
< 1 mm	1 (0.6)
DRM+	
< 5 mm	0 (0.0)
Follow-up (months)	
Mean (± SD)*	54.8 (13.1)
Median (range)*	52.0 (36.0–88.0)
Local recurrence overall	
No	153 (96.2)
Yes	6 (3.8)
Interval to local recurrence (months)	
Median (range)	19.2 (5.9–30.0)
Distant metastasis	
No	137 (86.2)
Yes	22 (13.8)
Interval to distant metastasis (months)	
Median (range)	6.9 (1.1–50.4)
Disease recurrence	
No	133 (83.6)
Yes	26 (16.4)
Interval to disease recurrence	
Months	8.2 (1.1–50.4)
Overall survival	124 (78.0)
Deceased	35 (22.0)
Interval to death (months)	
Median (range)	28.0 (0.5–61)

Numbers in parentheses are percentages, unless mentioned otherwise

*Mean/median range does not include diseased patients

### Risk factors for local recurrence

Univariate binary logistic regression analysis for local recurrence showed no significant difference for sex, obesity, low tumor, threatened mesorectal fascia, preoperative radiotherapy, (y)pT4 stage, (y)pN2 stage, positive CRM, incomplete mesorectum, intraoperative perforation, intraoperative purse-string failure, carbon dioxide embolus, synchronous metastasis, anastomotic leakage and reoperation. There was a significant risk for pathologic stage T3 or 4 tumors, RR 0.103 (0.012–0.904), *p* = 0.040, complications grade 3 or higher according to Clavien–Dindo RR 0.148 (0.026–0.844), *p* = 0.031 and presence of presacral abscess RR 0.077 (0.014–0.430), *p* = 0.003. The patient with intraoperative purse-string failure did not develop presacral abscess or local recurrence. Results of the univariate analysis for risk factors are summarized in Table 4.

**Table 4:** Univariate analysis of risk factors for local recurrence

	LR	Total	RR	95% CI lower	95% CI upper	*P* value
Sex						
Female	3	53	Ref			
Male	3	106	2.06	0.401	10.573	0.386
BMI > 25						
No	4	66	Ref			
yes	2	93	2.935	0.522	16.522	0.222
Low tumor < 4 cm from AV						
No	4	112	Ref			
Yes	2	47	0.833	0.147	4.713	0.837
MRF threatened on MRI						
No	4	125	Ref			
Yes	2	34	0.529	0.093	3.018	0.473
Preoperative radiotherapy						
No	2	47	Ref			
Yes	4	112	1.200	0.212	6.787	0.837
Pathologic stage T3–4						
No	1	102	Ref			
Yes	5	57	0.103	0.012	0.904	0.040
Pathologic stage T4						
No	6	157	Ref			
Yes	0	2		0.000		0.999
Pathologic stage N2						
No	5	146	Ref			
Yes	1	13	0.426	0.046	3.943	0.452
CRM+						
No	5	158	Ref			
Yes	1	1	0.000	0.000		1.000
Incomplete mesorectum						
No	6	155	Ref			
Yes	0	4		0.000		0.999
Intraoperative perforation						
No	6	157	Ref			
Yes	0	2		0.000		0.999
Purse-string failure						
No	6	158	Ref			
Yes	0	1		0.000		1.000
CO_2_ embolus						
No	6	158	Ref			
Yes	0	1		0.000		1.000
Synchronous metastasis						
No	5	152	Ref			
Yes	1	7	0.204	0.021	2.029	0.175
Complications CD 3 or higher						
No	2	120	Ref			
Yes	4	39	0.148	0.026	0.844	0.031
Anastomotic leakage						
No	5	149	Ref			
Yes	1	10	0.313	0.033	2.965	0.311
Presacral abscess						
No	3	145	Ref			
Yes	3	14	0.077	0.014	0.430	0.003
Reoperation						
No	3	123	Ref			
Yes	3	36	0.275	0.053	1.426	0.124

*BMI* body mass index (kg/m^2^), *AV* anal verge, *MRF* mesorectal fascia, *CRM+* involvement of the circumferential resection margins (< 1 mm), *CD* Clavien–Dindo

## Discussion

This study of 159 TaTME procedures for rectal cancer shows that TaTME is associated with low local recurrence rate; the 3-year local recurrence rate was 2.0% with complete follow-up and the 5-year local recurrence rate was 4.0%. The median time to local recurrence was 19.2 months (range 5.9–30.0 months). To the best of our knowledge this is the largest series with a complete and long follow-up of more than 3 years after TaTME.

The 3-year local recurrence rate in this study is relatively low compared to the laparoscopic TME long-term outcome data of the COLOR II, ALaCART and ACOSOG Z6051 trials, which show a 3-year local recurrence rate of 5% [6–8]. In accordance with previous literature, high tumor stage, severe postoperative complications and presence of a presacral abscess were risk factors for local recurrences [16]. A multivariate analysis was not possible due to the low number of events.

One of the potential benefits of TaTME for mid and low rectal cancer is a better specimen quality and better radicality. Incomplete mesorectum is a known risk factor for local and overall recurrence [17]. In our study, 97.5% of the specimens were of good quality, comparable to our previous study in which 100% of the specimens after TaTME were of good quality, while in the traditional laparoscopic group 80% were of good quality [18].

Although TaTME was introduced in 2010, ample data on long-term outcome are currently limited. In contrast, a considerable amount of case series describing single center experiences focus merely on short-term and pathological outcomes [19]. Although there is a growing interest in TaTME in rectal cancer surgery, it is still not widely implemented and concerns persist regarding the adequacy of oncological resection. Our study adds long-term outcome data to support the potential benefits of TaTME for mid and low rectal cancer: increased quality of the mesorectum, low number of positive CRM and corresponding low local recurrence rate.

Although the results from our study are encouraging, it only includes data from the two hospitals that started TaTME in The Netherlands which are high volume tertiary referral centers. The oncological results of widespread adoption of TaTME have not yet been demonstrated. Early adopters of TaTME recognized the high complexity of the procedure [20]. Therefore, several countries started a nationwide structured training program including proctoring to guarantee safe implementation of the procedure [21, 22]. The technique has a learning curve associated with substantial morbidity. Surgeons have to perform at least 40 cases to reach competency, based on acceptable morbidity or good pathologic outcome [9, 23]. Furthermore, higher volumes are associated with better outcome in terms of conversion, severe complications and quality of the mesorectum [3]. Our results do not support the concern that TaTME leads to an increased risk for local recurrence, as suggested by Norwegian data [10]. It is to be imagined that poor quality TaTME does negatively influence local recurrence rates. A review focusing on outcomes of TaTME in low volume centers was associated with a relatively high recurrence rate of 8.9% versus 2.8% in high volume centers [3].

This indicates that a steep learning curve might seriously hamper both short- and long-term outcome. Inadequate dissection, perforation and/or insufficient closure of the rectum before dissection all have the potential for tumor spill [24].

The Idea, Development, Exploration, Assessment and Long-term follow-up (IDEAL) framework aims to prevent surgical innovation from being implemented too early [25]. While the TaTME is still in the developmental stage and no global consensus and standardization has been reached, one could argue that the surgical community has proceeded to the adoption of this technique too early. This means exposing patients to potential intraoperative complications and short-term morbidity. Furthermore, long-term oncological safety of the technique must be established to avoid events comparable to the port-site metastasis setback seen in laparoscopic surgery [26]. The international TaTME registry is a useful instrument for capturing real-time data of the early adoption of TaTME and has signaled a 15.7% anastomotic failure rate [27]. The long-term follow-up data of the international registry are awaited, although the completeness of data will be a potential problem and source of bias.

Although the results of our study are promising, oncological safety after TaTME surgery remains to be proven in a multicenter international setting. The next crucial step in implementing this technique is an international randomized controlled trial such as the COLOR III trial, which is currently enrolling and is designed to assure high-quality evidence by implementing a pretrial showing surgical competency, central review of MRI, assessment of procedural video, re-evaluation of the specimen and obligatory upload and central review of MRI 3 years after surgery [28].

## Conclusions

TaTME is associated with relatively low local recurrence rate at 3 years and 5 years. This shows that in experienced hands with high volume, TaTME is safe and is associated with good long-term outcome.
